# Patients' Contributions to Hospitals

**Published:** 1888-05-19

**Authors:** W. Burdett-Coutts


### Patients' Contributions to Hospitals.

By Mr. W. Burdett-Coutts, M.P.

(Continued from p. 98.)

Before dealing with other countries it is appropriate that I here express my obligations to Mr. Henry Burdett, whose indefatigable work in this field has constituted him a prominent authority on all questions relating to hospitals. Mr. Burdett has made a special study of this subject, and his book, entitled "Pay Hospitals,"* first directed my own attention to it. Indeed, I imagine that the introduction of the system in most places where it is now existing in England has been due to him, and that he may fairly be called the pioneer of this reform. That book was published ten years ago, and it contains such a full account of the systems prevailing abroad that I need do little more than enumerate them; but I would commend the volume to the careful perusal of all who have not seen it. Mr. Burdett has lately been prosecuting some exhaustive inquiries on this and other subjects connected with hospitals in foreign countries, and has very kindly placed the information already received at my disposal; but I find on this subject of the Pay System there is little to add to the information contained in his book, except perhaps to say that it continues in all foreign countries to work smoothly and satisfactorily on the same lines as hitherto adopted. I am far from wishing to exchange our own for the foreign system; but they represent the two extremes. If we find that our own system is more precarious and difficult in proportion as it departs farther from theirs, and becomes more and more threatened every year with collapse, I submit the question, "Would it not be well and wise to incorporate with its present purely eleemosynary basis a certain amount of the self-sustaining and independent element?"

### Pay System in Foreign Countries.

From Mr. Burdett's interesting book I have obtained the following summary of the Pay System in foreign countries.

Broadly, it may be said, there is no absolutely free hospital abroad. The general system of hospital relief on the Continent is that the hospitals, with the exception of a few particular institutions, are under the control of the Municipalities or State, and are supported (as in France) by a revenue derived from charitable gifts invested and managed by a central authority usually connected with the Municipal or State Government, and by subsidies voted by the local Councils or State. The destitute poor are treated here as well as other patients, often well-to-do people, as the Continental system of living in flats renders it undesirable, if not impossible, to treat sickness at home. The system of payments will be found to prevail pretty generally in some form or other, all over the Continent, patients first being admitted to the hospital and then told off to a ward containing one, three, or more beds, according to the payment they are able to make.

Private hospitals are sometimes established, to which admission is free, as, for instance, the Hertford, at Paris—a memorial hospital endowed and intended only for English people; and the Maison Dubois, originally founded by a Frenchman to accommodate people of the better class who could pay a little for their treatment. This became very popular, and soon grew to a considerable building, but has now been absorbed by the Assistance Publique, and is one of the hospitals of Paris worked under the general system. The following are

### Typical Instances.

The Maison Municipale de Sainté has 600 beds; but though established and maintained out of the funds of the Administration de l'Assistance Publique, is not, strictly speaking, a Hospital, inasmuch as no medical teaching is carried on, and no clinical or other instruction is given there. A permanent medical staff is attached to the Maison, and any patient may call in consultation any honorary physician or surgeon who may be connected with the large Parisian hospitals, but at his own expense and with the consent of the medical director. Friends are admitted as visitors, and for certain fixed payments can be provided with accommodation in the hospital itself, an arrangement especially valuable in the case of children. The payments, which vary from four to twelve francs a-day, include visits, consultations, and operations, diet, dressings, medicine, linen, fires, and baths. For twelve francs a separate room for the accommodation of a nurse or friend is provided. A small private room costs eight francs a-day; a double-bedded room costs six francs. For five francs a bed is provided in a room with three others, for four and a-half francs in a four-bedded room, and for four francs in a still larger room. All payments are made in a`vance. This hospital paid until 1860, but in that year the system of small rooms was first introduced, and as these are comparatively seldom occupied there has been a marked deficiency since

### Germany and Austria.

Their systems are identical. In both countries it is the custom for eminent surgeons to be associated with private clinics, or hospitals, of which they are often the proprietors. There are also similar establishments belonging to a lay manager or owner, but, in all, each patient pays according to th accommodation and attention which his case necessitates. Of course the charge often bears a proportion to the fees paid for consultation, etc., which are regulated by the standing and reputation of the medical attendant. Professor Volkman, of Halle, has five of these institutions under his own management and direction, and one of his rules is that every operation shall be performed in the operation theatre of the public hospital.

In Berlin there is more than one public pay hospital, the chief of them being the Augusta, where both in and outpatients pay according to their means. In every grade the terms are inclusive, and for one payment the best of medical treatment, diet, and nursing is provided. By far the larger number of patients occupy beds in a general ward containing from two to thirty beds. The charges vary from two to eight shillings per diem. All the members of the medical staff are paid for their services. All classes of society are found in the wards. Two other hospitals in Berlin admit a considerable proportion of pay patients—the Bethanien and the Jewish Hospitals—the former the property of a society of deaconesses or sisters. The Jewish Hospital is kept up by the Jews of Berlin. If a Jew is too poor to pay, he is admitted to a free bed, but unless be can prove his poverty he has to pay from two shillings and twopence to seven shillings and sixpence per day, the latter sum ensuring a separate room. Of the German hospitals generally, it may be said that all, or very nearly all, admit patients on payment, and although free beds are not uncommon, still the thrifty portion of the German public who resort to the hospital prefer to pay according to their means for the treatment they receive.

### Spain and Italy.

In Spain all the hospitals take paying patients, but those managed by sisters of charity are practically free. Generally, if it is proved that the patient is poor and can contribute nothing to his support, he is placed in one of the large wards, and is paid for by the local authorities at such a rate as they may consent to give to the hospital authorities. On the other hand, patients who desire to preserve their self-respect

* "Pay Hospitals of the World." London: J. and A. Churchill and a certain privacy, are admitted on payment of a sum of from two shillings to two shillings and sixpence per day. Seamen falling ill in a Spanish port are sent into the Spanish Seaport Hospital and paid for by them at from one shilling and eightpence to two shillings per day. Probably the most typical instance of a remunerative hospital has been established in Italy. Every Italian city has one of these institutions, but the Casa di Salute at Milan deserves prominence both from its size and antiquity. It is managed by a society which has now been incorporated with the consent of the Government, but owes its origin to a legacy bequeathed by a gentleman about 1830 for the purpose of its establishment. It was originally worked for twenty years as an experiment, and at the end of that period, the results being considered satisfactory, the amount of capital was increased. The capital of the society is divided amongst a hundred shareholders, each possessing one share of fifty pounds. This has been invested for the most part in improving and maintaining the buildings. Each patient costs about six shillings per diem, and the scales of payment are six shillings, seven shillings and sixpence, and nine shillings; and after payment of all necessary expenses, and a deduction from the net profits of ten per cent. for wear and tear, and twenty-five per cent. to form a reserve fund, there has invariably remained a balance sufficient to pay a dividend of five per cent. to the shareholders. Payment is demanded in advance.

Sweden and Norway.

The best system of hospital relief to be found anywhere in Europe is probably that adopted by the Government in the State Hospital, Christiania. The hospital contains 238 beds, the number being frequently found inadequate. In connexion with the hospital is a bathing establishment of considerable extent, which is used by the public at large, and produces a goodly portion of the income of the hospital. The rest of the income is derived mainly from the payments made by the patients, and the institution is almost entirely self-supporting. As, however, it is regarded as a Government institution, the directors annually submit a budget for the consideration and approval of Parliament, their calculations being based upon the number of patients, the number of beds occupied, and the daily payments of each patient during the previous year. Any difference between the estimated income and the estimated expenditure is voted by the Chamber. Every inmate of this hospital becomes a pay patient, because the Government recognises the economy of working the voluntary charities and the poor-law charities by means of the same central administration. The poor-law authorities are enabled, on payment of a fixed sum, to send their poor-law cases for treatment to the hospital, by which means the medical staff is always provided with sufficient material for the clinical instruction of the students No pay patient, except a poor-law case, is subjected to any other examination than that necessary for diagnosis.

Three classes of pay patients are admitted—first, the poor-law cases, who occupy wards with from four to ten beds, and pay three shillings per day; secondly, patients occupying wards containing two or at the outside three beds, and paying three shillings and eightpence per day; and, thirdly, those having each a separate room, with special nursing and attendance, and paying six shillings and eightpence a-day. Mr. Burdett says: "It is an interesting fact, and shows the advantage of harmonising the working of all institutions, which have for their object the relief of suffering, that the efforts of the Norwegian and Swedish Government have resulted in the establishment of a National Hospital, where 130 beds are provided for poor-law cases, in addition to 110 for pay patients." This plan has enabled the Government to relieve their sick paupers free of cost to the State. In other words, the State Hospital, Christiania, is practically self-supporting. *(To be continued.)*

